# Perceived stress among university students in Oman during COVID-19-induced e-learning

**DOI:** 10.1186/s43045-021-00131-7

**Published:** 2021-08-04

**Authors:** Mustafa Malik, Sarfaraz Javed

**Affiliations:** 1 Department of Management, University of Nizwa, Nizwa, Oman; 2 Department of Commerce, Aligarh Muslim University, Aligarh, India

**Keywords:** Stress, Students, Higher education, COVID-19, Oman

## Abstract

**Background:**

Higher education institutions across the world moved to e-learning in response to the disruptions caused by the pandemic. While e-learning has an advantage for students that they can attend to their courses from anywhere at their convenience, yet the sudden disruptive shift to e-learning during the pandemic saw students facing many challenges, which had strong ability to induce mental health issues among the students. This study aimed at examining the impact of COVID-19-induced e-learning on the university students’ stress perceptions in Oman.

**Results:**

A total of 966 usable responses were received. The results showed that 96.9% (moderate stress = 82.5% and high stress = 14.4%) reported having experienced stress through e-learning during COVID-19. The results further indicated that there was a significant negative correlation between perceived stress and the students’ academic performance. The family support and institutional support were observed to have a significant effect on students’ stress perception.

**Conclusions:**

Students are away from the physical campuses over a year now, continuing their courses and programs online. The numerous challenges they are facing through e-learning, together with the prevailing uncertainty, have intensified stress among them. The continued stress over time will not only affect their academic performance, but their mental and physical health as well, as the stress has proven to be one of the major causes of various physical and mental disorders. While e-learning seems to emerge as a new normal, the students require proper attention, help, and support from their families and institutions. The institutions should revisit their online courses and program delivery mechanisms, methods, and practices to ensure that students are not over stressed.

## Background

The spread of novel coronavirus (COVID-19) disrupted all economic and social activities around the world. Higher education has been one of the worst hit sectors. Being intensively interactive in nature, this sector saw a comparatively early and complete shutdown across the globe. In Oman, the government announced the closure of all educational institutions by mid of March 2020. Responding to the pandemic and the subsequent advisories by regulating bodies to suspend on-campus academic activities, higher education institutions (HEIs) switched to online mode to deliver academic programs. Like elsewhere, most of the HEIs in Oman had no prior experience of delivering programs completely online and therefore, applied varied approaches depending on their size, governance models, and disciplinary differences [1]. Over more than a year in pandemic now, HEIs in Oman are continuing their programs online. With many improvements and innovations in online teaching and learning process over the past 1 year, students have started adapting to this new normal.

However, weakness of online teaching infrastructure, inexperience of teachers regarding new technologies, information gap, and complex home environment still exist [2]. Students, through COVID-19-induced online education are facing numerous challenges, such as *instructional* [3] *technological, and technical* [3–5]; *social and family atmosphere* [5]; *home confinement* [6, 7]; *emotional and psychological* [3, 8]. Furthermore, many students do not find a suitable space in their homes for effective learning and do not have access to sufficient hardware or internet which constrains their home learning [9].

Though online education for HEIs is not anything new, as many HEIs have been offering full courses and/or full programs online, yet students enrolled in full online instruction-based programs, who were not familiar with such experiences faced a system shock [10].

All these challenges faced by the students in online education during COVID-19 have a strong ability to induce mental health issues among the students. One commonly experienced mental health issue by university students is the academic stress, which is mostly due to the students’ apprehension of loss of grades and fear of failure [11]. Fear of lower performance and delay in completion of studies are also the reasons to induce stress among students during COVID-19. Many studies conducted during last 1 year focusing on university students’ mental health during COVID-19, such as [12–16] concluded that COVID-19 had a negative impact on the mental health and wellbeing of the university students. Besides earlier studies such as [17–19] have also shown that uncertainties due to public health emergencies, such as H1N1 influenza, Ebola, SARS, and MERS, caused negative psychological effects on university and college students. Furthermore, the negative psychological impacts are aggravated if the public emergencies accompany with home confinement [6, 7], which is what happened across the globe, during COVID-19.

In the context of Oman, there are very few studies conducted regarding the impact of COVID-19 on the mental health of people and these are mostly related to the *youth in general* [20], *general population* [21], and *health workers* [21]. Regarding the mental health of university students during COVID-19 in the context of Oman, we did not find any study except for one by Alqassabi et al. [22], published in Arabic language. The available literature indicates that there is little being published on impact of COVID-19-induced e-learning on the stress perceptions of university students in Oman. While there is considerable literature on this subject available in the Western and Eastern contexts, these cannot necessarily be generalized to Arab world considering that its culture, social structure, and social norms vary greatly from the Western and Eastern cultures.

Thus, the present study aimed at examining the impact of COVID-19-induced e-learning on the university students’ stress perceptions in Oman, thereby filling the gap in the literature that exists in the Oman context on the topic under study. The results of this study provide insights to the higher education educators, administrators, and policy makers to enhance e-learning implementation in a way that ensures mental and psychological wellbeing of the students.

## Methods

### Sample

This study was undertaken at the University of Nizwa, Oman. It is the second largest and the only non-profit private university in the country. The university comprises of four colleges namely College of Arts and Sciences (CAS), College Economics, Management and Information Systems (CEMIS), College of Pharmacy and Nursing (CPN), and the College of Engineering and Architecture (CEA). A total of 5269 students were registered across all undergraduate programs in the university for Fall 2020 [CAS = 2556 (48.5%); CEMIS = 1569 (29.8%); CPN = 552 (10.5%); and CEA = 592 (11.2%)] (*data received from Center for Information Systems of the University by email on the request of the corresponding author*). A self-administered questionnaire was sent online to all the registered students. A total of 966 [CAS = 573 (53.5%); CEMIS = 273 (28.3%); CPN = 74 (7.7%); and CEA = 102 (10.6%)] completed responses were received. The figures show that the percentage of respondents from each college is fairly proportionate to the percentage of total students in each college, thus the sample is fairly representative of each college of the university under study.

### Measures

#### Perceived Stress Scale (PSS-10)

To assess the stress as perceived by the students, the Cohen’s Perceived Stress Scale (PSS-10 [23];) was used. It is a self-reported questionnaire that measures the degree to which an individual appraises situation in his or her life as stressful. The scale consists of 10 items including 6 positively phrased and 4 negatively phrased items. Each item is rated by respondents on a 5-point scale ranging from ‘*never* (0)’ to ‘*very often* (4)’. The scores for negatively phrased items are reversed and then the scores for all 10 items are added for each individual. The individual scores on PSS can range from 0 to 40 with higher scores indicating higher perceived stress (‘*low stress* = 0–13’, ‘*moderate stress* = 14–26’, and ‘*high stress* = 27–40’). The scale has been used in numerous studies and has shown good reliability. Lee [24] in his review paper on the use of PSS-10 reported that Cronbach’s alpha was evaluated at > 0.70 in all 12 studies his research referred to. For the present study the Cronbach’s alpha for PSS-10 scale was 0.76.

#### Stressors

Potential factors leading to stress among the university students were derived from the literature [25–28] and after informal discussion with the students. The students’ inputs were very beneficial as it helped the authors to identify factors that were more relevant to online education and in the present pandemic situation. A total of 14 potential source of stress were identified. These were grouped into three categories as academic stressors (7 items), psychological stressors (4 items), and social stressors (3 items). For each potential stressor, the respondents were asked to show their agreement or disagreement on a 5-point Likert scale ranged between ‘*highly agree* as 5’ and ‘*highly disagree* as 1’. The 14 stressors used in this study showed an overall Cronbach’s alpha of 0.78.

### Procedures

The survey for the present research was conducted online using Google forms, with the undergraduate students registered for Fall Semester 2020 at the University of Nizwa in Oman. A bilingual (Arabic and English) questionnaire was sent to the students through their university email and was also posted on the University’s Moodle home page with the help of Center for Information Systems of the University to ensure wider participation. The survey was open between 6 December 2020 and 31 December 2020 which corresponded to week 13 to week 16 of the academic semester (last 4 weeks before the final examination). During this period, three email reminders were sent to all the students for completing the survey. In the introductory part of the questionnaire, the purpose of this research was briefly explained. Students were informed that the participation in the survey was completely voluntary and were asked to express their consent before proceeding to respond to the questionnaire. To conduct this study, we received an ethical approval from the Human Ethics Committee in the Office of the Vice Chancellor for Graduate Studies, Research and International Relations, University of Nizwa through its letter (EC Ref. No.: HREC-12-2020) on 17 November 2020.

### Statistical analysis

The statistical analysis was performed using the Statistical Package for Social Science (SPSS-20). Apart from descriptive statistics, means, standard deviations, and Pearson correlation was used to analyze and interpret the data and write the results.

## Results

### Descriptive statistics

As presented in Table 1, a total of 966 student-participants responded to the survey. By gender, the respondents comprised of 84.4% females (*n* = 815) and 15.6% males (*n* = 151). The female:male ratio of respondents was proportionate to the female:male ratio of the total student population (*n* = 5269) registered for Fall 2020 in the university (females = 4603, 87.3%) and males = 666, 12.6%).

**Table 1:** Demographic characteristics of the respondents and mean score on PSS-10 (*n* = 966)

				PSS10	
		**Number**	**%**	**Mean**	**SD**
Gender	Male	151	15.6	21.0	4.3
	Female	815	84.4	22.2	4.2
Age	≤ 20	424	43.9	21.9	4.3
	21–25	420	43.5	22.2	4.1
	≥ 25	122	12.6	21.3	4.3
Marital status	Married	133	13.8	22.6	4.2
	Unmarried	833	86.2	21.3	4.3
Place of residence	Rural	578	59.8	21.9	4.6
	Urban	388	40.2	22.1	4.7
Family type	Nuclear family	480	49.7	21.5	4.4
	Joint family	486	50.3	22.4	4.1
Degree level	Bachelor	689	71.3	22.2	4.3
	Diploma	277	28.7	21.3	4.1
Study year	Year 1	292	30.2	21.4	4.3
	Year 2	160	16.6	22.3	4.3
	Year 3	184	19.0	21.7	4.3
	Year 4 and above	330	34.2	22.5	4.2
CGPA	≤ 2.0	99	10.2	21.6	3.6
	2.00–2.49	179	18.2	21.4	4.0
	2.50–2.99	248	25.7	22.0	4.0
	3.00–3.49	262	27.1	22.6	4.4
	3.50–4.00	178	18.4	21.6	4.8
College	CEMIS	273	28.3	21.9	3.6
	CAS	517	53.5	21.9	4.5
	CPN	74	7.7	21.9	3.6
	CEA	102	10.6	22.5	4.5

### Perceived stress among respondents

As presented in Table 2, the results of this study showed that the mental health of students is negatively affected during the pandemic. Of 966 students surveyed, 936 (96.9%) reported having experienced stress (moderate stress = 82.5% and high stress = 14.4%). There were only 30 (3.1%) students who reported having experienced low stress.

**Table 2:** Stress among students on PSS 10 scale

Stress level	Number (***N*** = 966)	(%)
**Low** *(PSS Score 0–13)*	30	3.1%
**Moderate** *(PSS Score 14–26)*	797	82.5%
**High** *(PSS Score 27–40)*	139	14.4%

When comparing mean stress scores between groups, the stress score varied between 21.0 and 22.6 among all categories of respondents, thereby showing that on an average all the respondents experienced a high-moderate stress. Similarly, female students (*M* = 22.2, SD = 4.2) showed higher symptoms of stress compared to male students (*M* = 21.0, SD = 4.3), and the mean stress score observed for married students (*M* = 22.6, SD = 4.3) was higher as compared to unmarried students (*M* = 21.3, SD = 4.2). Similarly, students from urban areas were comparatively more stressed than those living in rural areas, students living in joint families were comparatively more stressed than those living in nuclear families, students enrolled in bachelor’s degree programs were comparatively more stressed than their counterparts in diploma degree programs. While comparing mean stress score of students based on the year of study, the students studying in Year 2 and Year 4 showed comparatively higher stress than those studying in Year 1 and Year 3. Based on the CGPA of the students, the students falling at the middle of the CGPA continuum (2.50–3.50) showed comparatively higher stress than those with lower (≤ 2.0–2.5) and higher (3.5–4.0) CGPA. When comparing the students’ mean stress scores based on the college they belonged to, the engineering students showed a comparatively higher stress (CEA, *M* = 22.5, SD = 4.5) than students of other three colleges (CEMIS, *M* = 21.9, SD = 3.6; CAS, *M* = 2.19, SD = 4.5 and CPN, *M* = 21.9, SD = 3.6).

### Perceived institutional and family support

Organizational (institutional) support [29] and social (including family) support [30] is generally perceived to have positive influence on peoples’ health and wellbeing. In academic settings, the institutional support may refer to the academic and non-academic support and services provided by the institutions to its students. In the context of e-learning during COVID-19, it may refer to the online academic support available to the students from their mentors, advisors, and teachers as well as from the institution’s academic support services such as online learning support, technical and technological support, communication with students, and response to students’ academic concerns. On the other hand, family support during COVID-19 may refer to the support provided by a student’s family in terms of time and space and family responsibilities etc. The respondents (students) were asked to evaluate the institutional and family support during COVID-19-induced e-learning as *poor*, *moderate*, or *good*. The results of this study, as presented in Table 3, showed that 95.2% (good = 66.9% and moderate = 28.3%) students perceived family support as favorable compared to 72.5% (good = 21.7 and moderate = 50.8%) perceiving institutional support as favorable. A considerable percentage of students (27.4%) perceived the institutional support as poor.

**Table 3:** Students perceived family support, institutional support and academic performance (*N* = 966)

	Poor	Moderate	Good	Mean	SD
Perceived family Support	47 (4.9%)	273(28.3%)	646 (66.9%)	2.62	.577
Perceive institutional support	265 (27.4%)	491(50.8%)	210 (21.7%)	1.94	.699
	**Poor**	**Same**	**Better**	**Mean**	**SD**
Perceived academic performance compared to pre-COVID19	377(39%)	323 (33.4%)	266 (27.6%)	1.89	.808

### Self-reported academic performance

One of the major factors leading to stress among the students and widely reported in the literature is the fear of losing grades and poor academic performance. In this study, the students when asked to compare their academic performance with that of pre-COVID-19 period, 39% reported poor academic performance compared to 27.6% reporting their academic performance has improved, and the rest 33.4% said that they have not observed any change in academic performance (Table 3).

### Stress factors

Various factors contributed to the stress experienced by students in online education during the pandemic. These factors were categorized as (a) academic factors, (b) psychological factors, and (c) social factors. Table 4 presents ranking of perceived stress factors based on their mean. Among the academic factors, while all factors have been perceived as contributing to the students’ stress with the mean varying between 3.63 (lowest) and 4.12 (highest), *increased number of exams* during online distance learning was perceived as the strongest factor leading to stress among students (mean = 4.12, SD = 0.98) and *sufficiency of learning materials provided by instructors* was ranked at lowest (mean = 3.89, SD = 1.01). Among the psychological factors, again all the factors were perceived as contributing to the stress among the students with the mean score varying between 4.33 (highest) and 3.50 (lowest). *Constant fear of losing grades* (mean = 4.33, SD = 0.93), and *worry about performance* (mean = 4.27, SD = 0.96) were ranked as top two psychological stress factors. Among the social factors, concern for family sufferings, *my family is suffering due to my over engagement in academic work* (mean = 3.91, SD = 1.06) was ranked at highest.

**Table 4:** Ranking of stress factors by mean (*N* = 966)

***Academic factors***	Mean	SD
1. I have to attend to more exams than I used to attend before COVID-19	4.12	0.98
2. Online education during COVID-19 has increased my academic workload	3.96	1.03
3. I am facing technical difficulties (no or poor access to required technology)	3.96	1.08
4. Online education has made it difficult to manage my time	3.89	1.09
5. I am facing difficulty understanding course contents through online educational platforms	3.88	0.99
6. My performance in exams has decreased during COVID-19	3.83	1.09
7. Learning materials provided by the instructors are not sufficient.	3.63	1.01
***Overall academic***	**3.89**	**0.75**
***Psychological factors*** **(*N*** **= 966)**		
1. I have constant fear of losing grades	4.33	0.93
2. I am most of the time worried about my performance	4.27	0.96
3. I worry about my health	4.13	1.05
4. I am not able to concentrate during online lectures	3.50	1.09
***Overall psychological***	**4.06**	**0.69**
***Social factors*** **(*N*** **= 966)**		
5. My family is suffering due to my over engagement in academic work	3.91	1.06
6. I get very little support from my family to manage my academic requirements	3.74	1.17
7. My teachers are less cooperative in solving my academic problems	3.50	1.06
***Overall social***	**3.72**	**0.82**

### Correlation among variables

This study showed that the academic performance was negatively correlated with the overall perceived stress (*r* = − .349, *p* < 0.01), negatively correlated with perceived academic stressors (*r* = − .511, *p* < 0.01), negatively correlated with perceived psychological stressors (*r* = − .383, *p* < 0.01), and also negative correlated with perceived social stressors (*r* = − .100, *p* < 0.01) (Table 5). Furthermore, when comparing the correlation of academic performance with the perceived stressors, it was observed that perceived academic stressors had a significantly higher negative correlation with academic performance (*r* = − .511, *p* < 0.01) compared to perceived psychological stressors (*r* = − .383, *p* < 0.01) and perceived social stressors (*r* = − .100, *p* < 0.01).

**Table 5:** Correlation among family support, social support, perceived stress, and academic performance

		1	2	3	4	5	6	7
1. **AcPer**	*r*	1	.262**	.351**	− .349**	− .511**	− .383**	− .100**
	*Sig.*		.000	.000	.000	.000	.000	.002
2. **FamSup**	*r*	.262^**^	1	.331**	− .203**	− .277**	− .209**	− .064*
	*Sig.*	.000		.000	.000	.000	.000	.047
3. **InsSup**	*r*	.351^**^	.331**	1	− .296**	− .401**	− .343**	− .028
	*Sig.*	.000	.000		.000	.000	.000	.388
4. **PerStress**	*r*	− .349^**^	− .203**	− .296**	1	.415**	.409**	.126**
	*Sig.*	.000	.000	.000		.000	.000	.000
5. **AcaStress**	*r*	− .511^**^	− .277**	− .401**	.415**	1	.691**	.389**
	*Sig.*	.000	.000	.000	.000		.000	.000
6. **PsyStress**	*r*	− .383^**^	− .209**	− .343**	.409**	.691**	1	.452**
	*Sig.*	.000	.000	.000	.000	.000		.000
7. **SocStress**	*r*	− .100^**^	− .064^*^	− .028	.126^**^	.389^**^	.452^**^	1
	*Sig.*	.002	.047	.388	.000	.000	.000	

**p = < 0.01 (2-tailed)

*p = < 0.05 level (2-tailed)

*r* = Pearson correlation coefficient, *Sig.* level of significance, *AcPer* academic performance, *FamSup* family support, *InsSup* institutional support, *PerStress* perceived stress (on PSS10 Scale), *AcaStress* academic stressors, *PsyStress* psychological stressors, *SocStress* social stresssors

## Discussion

The results of this study showed that COVID-19-induced online learning had a negative impact on the mental health of university students in terms of perceived stress, which was reported as moderate to high by 96.9% of the respondents. These results are consistent with most of the studies conducted during last 1 year such as 12], [13–16]. Higher stress among university students has been, most often, related to lower academic performance [31–33]. Through this study, it was observed that the students’ perceived academic performance had a negative relationship with their overall perceived stress on PSS10 (*r* = − .349, *p* < 0.01).

Furthermore, the results showed that perceived family support has a negative relationship with perceived stress (*r* = − .203, *p* < 0.01) and positive relationship with the academic performance (*r* = .262, *p* < 0.01). Deihl et al. [34] observed that people who perceived lower support from family and friends were emotionally less positive and hence more stressed. Moreover, Solberg [35] opined that social support moderates the relationship between stress and distress, which means that people who perceive higher social (family) support report lower distress. It is important to mention here that Cohen et al. [[30], p. 4] defined social support as ‘a process through which the social relationships promote health and wellbeing’.

While many studies have studied impact of social support on students’ stress, the impact of institutional support on students’ perceived stress has received little focus, especially in the studies those have been conducted during the pandemic. Since during the pandemic there was an abrupt shift from the physical classroom to online platforms, the institutional support to the students, in terms of advising systems, instructions, program progress tracking, career planning, and technology support, had a considerable impact on how comfortable or not they were with the online learning. This study showed that the students’ perception of institutional support has significant negative relation with students’ perception of stress (*r* = − .296, *p* < 0.01). This demonstrates that higher education institutions’ student support systems have a potential to mediate the stress, especially the academic stress, which in this study is significantly negatively correlated to perceived institutional support (*r* = − .401, *p* < 0.01) and academic performance (*r* = − .511, *p* < 0.01). Pascoe et al. [36] argue that enhancing student support in the education setting may improve the mental health of young people.

When comparing mean stress scores between groups, the stress score varied between 21.0 and 22.6 among all categories of respondents, thereby showing that on an average all the respondents experienced a high-moderate stress. These results are similar to other studies conducted during the pandemic such as [3, 5–9].

When comparing the mean stress scores (see Table 1), female students (*M* = 22.2, SD = 4.2) showed higher symptoms of stress compared to male students (*M* = 21.0, SD = 4.3). The mean stress score observed for married students (*M* = 22.6, SD = 4.3) was higher as compared to unmarried students (*M* = 21.3, SD = 4.2). Similarly, students from urban areas were comparatively more stressed than those living in rural areas, students living in joint families were comparatively more stressed than those living in nuclear families, and students enrolled in bachelor’s degree programs were comparatively more stressed than their counterparts in diploma degree programs. While comparing mean stress score of students based on the year of study, the students studying in Year 2 and Year 4 showed comparatively higher stress than those studying in Year 1 and Year 3. Based on the CGPA of the students, the students falling of the middle of the CGPA continuum (2.50–3.50) showed comparatively higher stress than those with lower (≤ 2.0–2.5) and higher (3.5–4.0) CGPA. When comparing the students’ mean stress scores based on the college they belonged to, the engineering students showed a comparatively higher stress (CEA, *M* = 22.5, SD = 4.5) than students of other three colleges (CEMIS, *M* = 21.9, SD = 3.6; CAS, *M* = 2.19, SD = 4.5 and CPN, *M* = 21.9, SD = 3.6).

## Conclusions

The spread of COVID-19 affected every aspect of human life. Its impact has been unprecedented. The closure of universities for on-campus face-to-face teaching and learning forced higher education institution across the globe to deliver programs online. Initially thought to be a temporary arrangement to deal with the crises, online education is emerging as a new normal. In Oman, as of now, higher education institutions are closed for on-campus teaching and learning for over a year, and courses and programs are delivered either completely online or through blended learning mode. The recent resurge in COVID-19-infected cases in the country has once again triggered a sense of uncertainty on the opening of universities for on-campus activities. Sahu [14] and Tayefi [37] observed that consistent closure of universities for on-campus classes triggered a sense of uncertainty among the students about their academic and professional career and intensified among them persistent mental health challenges. Given this context, the present study was conducted to investigate the impact of the COVID-19-induced online learning on university students’ psychological health in Oman. The study further investigated the relationship between the students’ perceived level of stress and their perceived family support and institutional support, as well as their academic performance.

The study showed the during the online teaching and learning induced by the pandemic, students in general have experienced a moderate to higher stress. The students’ mental health is significantly affected by the support they perceive from their family and the institution, as these were observed to be negatively correlated to the perceived stress by students. Although, academic, social and psychological stressors were observed to have negative impact on students’ mental health (perceived stress), yet academic stressors were observed to have comparatively higher negative correlation with students’ perceived stress as compared to psychological and social stressors, during COVID-19-induced online learning.

Various studies have shown that mental health of populations is significantly affected when faced with public health emergencies, and university students are no exception to this fact. The COVID-19 pandemic has been more catastrophic than any other public health emergency witnessed in the recent past. University students have been out of the physical campuses over a year now, and courses and programs are, more or less, completely delivered online, and there seems to be no end to it, at least not for the next six months to 1 year.

If the students are perceiving continued stress over time, it will not only affect their mental health but physical health as well, as the stress has proven to be one of the major causes of various physical and mental disorders such as hypertension, depression, diabetes, asthma, obesity, and cardiovascular diseases [38–41]. Hence, the students require proper attention, help, and support from their families and institutions [12]. The institutions should revisit their online courses and program delivery mechanisms, methods, and practices to ensure that students are not over stressed, particularly in terms of number of assessments, academic workload, and technical difficulties they face.

## Limitations

This study focuses on stress perceived by university students in Oman during COVID-19-induced online learning. Although the results of this study provide significant insights on the students’ perceived stress during online learning, as not many studies are available on the subject in the context of Oman, yet the findings of this study cannot be generalized for the entire country as the study was conducted on the students in a specific university. Further studies could be conducted involving a wider cross-sectional sample across higher education institutions in Oman to generalize the findings.

## Data Availability

All the data and materials relevant to this study are available with the corresponding author and can be made available on a reasonable request.

## Abbreviations

COVID-19: Coronavirus disease 2019
HEIs: Higher education institutions
MERS: Middle East respiratory syndrome
PSS-10: Perceived stress scale-10
SARS: Severe acute respiratory syndrome
SPSS: Statistical package for social science
